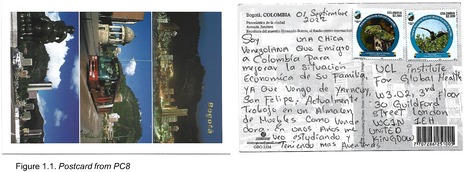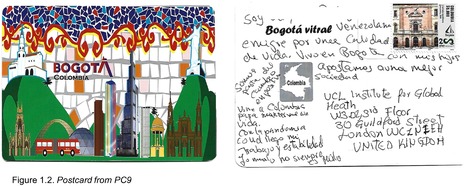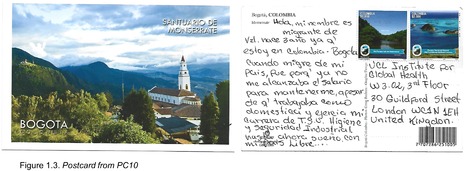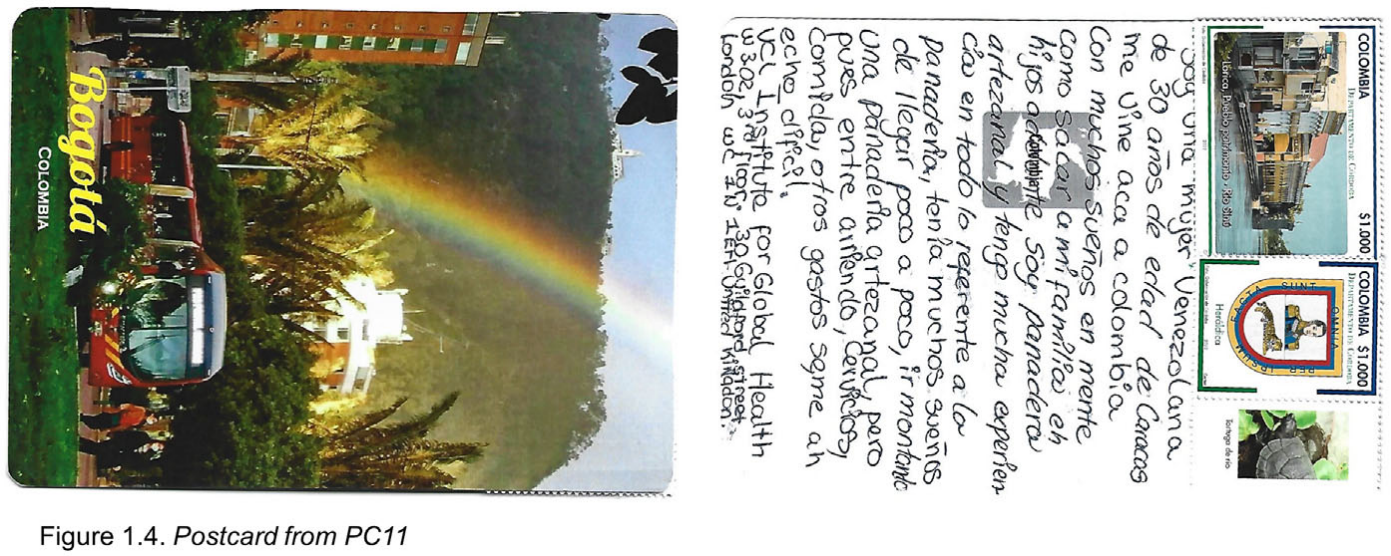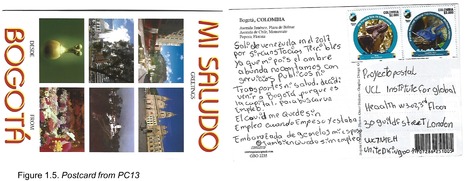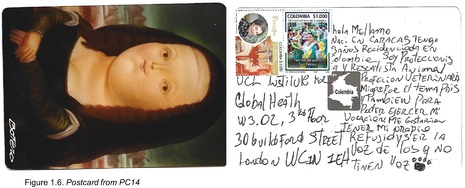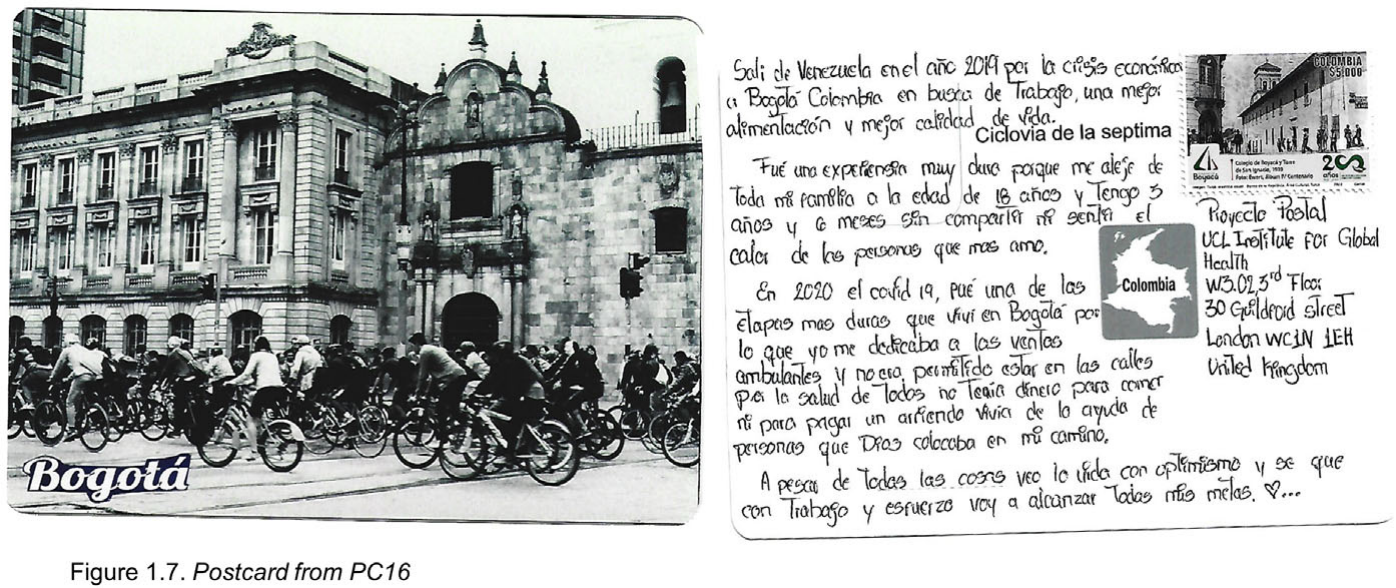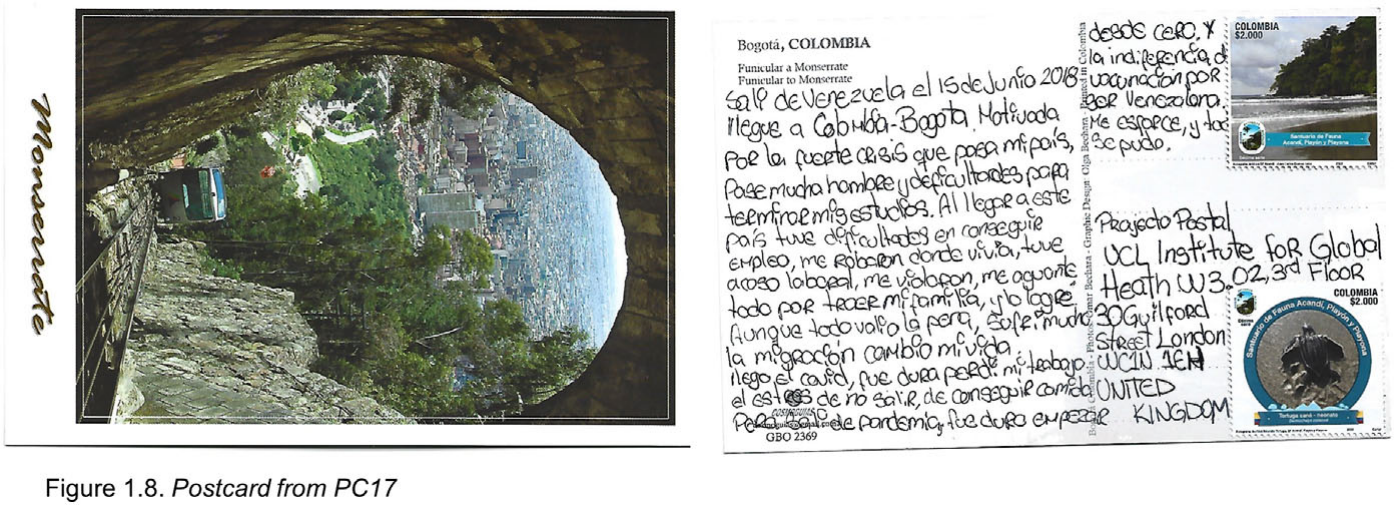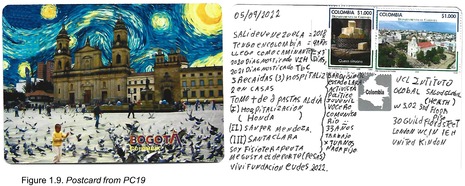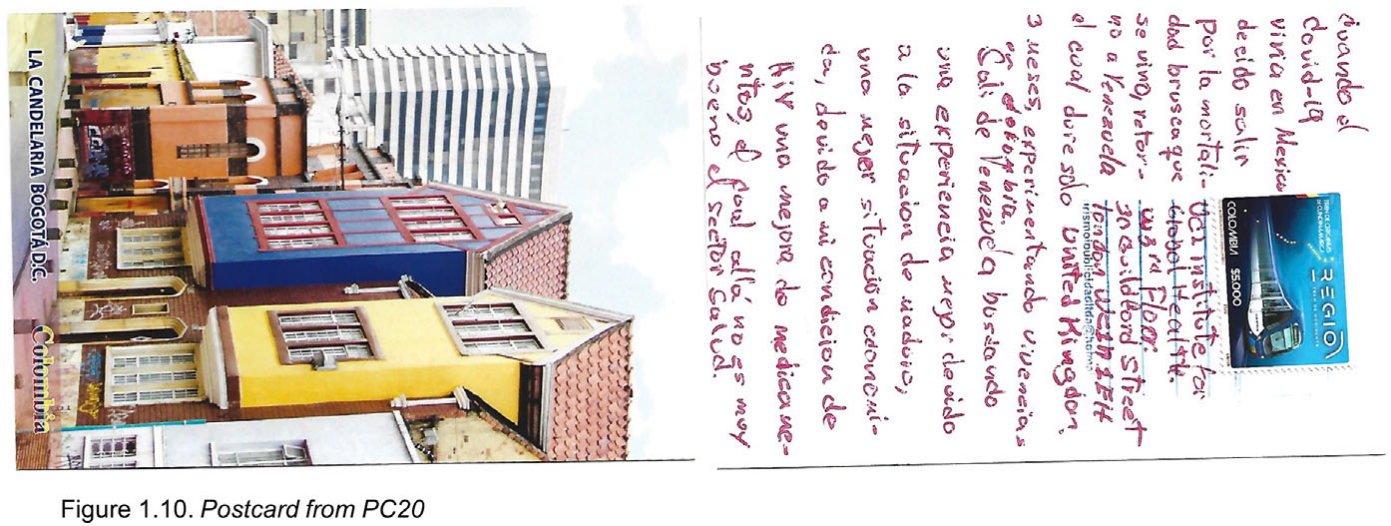# Postcards Post‐Petroleras: Exploring Collaborative Ethnography via Mail from the Venezuelan Diaspora

**DOI:** 10.1111/blar.13554

**Published:** 2024-04-15

**Authors:** Rebecca Irons

**Affiliations:** ^1^ University College London UK

**Keywords:** migration, pandemic, participatory ethnography, postcards, Venezuela, visual data

## Abstract

Postcards, as ‘travelling communication devices’, have been identified as excellent tools for their ability to collapse ‘the field’ in a new, visually experiential way. Though often presented as a (post)colonial medium that has exoticized ‘the other’, they may also be able to give snapshots of diverse biographies otherwise silenced, and can therefore be utilised as a form of collaborative ethnography. This paper analyses postcards sent from Venezuelans living in Bogota during the pandemic. It will suggest that when migrants express lived‐experience via postcards, the coloniality of the medium is challenged and reimagined as collaborative ethnography.

Venezuelans have dispersed across the globe, forming a significant diaspora that a variety of scholars, such as those in this special issue, are slowly developing a body of research with and on. The country with the largest migrant intake at 2.9 million migrants is Colombia (R4V, 2023), where the participants in this study reside. Whilst settled migrant populations may be accessible via established networks and online mediums, other groups such as the *caminantes* (see Irons and Brown, this volume), those in transit, and informal workers living on the margins have been harder to reach through traditional fieldwork methods (Pavón Hernández and Ramírez Moncaleano, 2022). This situation was only exacerbated by the COVID‐19 global pandemic; a time when online and virtual methods became the norm for many researchers in their respective lockdowns (Addeo et al. 2021; Abad Espinoza, 2022). Yet, this research method was an issue amongst those with digital poverty, where individuals may not have access to cellular phones, laptops and/or sustained mobile data availability, which makes it, therefore, difficult to work with and understand the experiences of such groups, especially when it comes to refugees and migrants (Alam and Imran, 2015). As a creative, participatory solution to working collaboratively with the moving Venezuelan diaspora, the project addressed in this paper eschewed online methods of data collection for their impracticality given the circumstances. Instead, Venezuelan migration stories were brought from Colombia to London via the post.

Postcards, as ‘travelling communication devices’, have been identified as excellent pedagogical tools for their ability to collapse ‘the field’ and ‘the classroom’ in a new, visually experiential way (Gugganig and Schor, 2020a). Though postcards are often presented as a (post)colonial medium that has exoticised ‘the other’ (Gugganig and Schor, 2020b), they may also be able to give snapshots of diverse biographies otherwise silenced (Levell, 2020) and, as Harris and Rawlinson (2020) suggest, can therefore be utilised as a form of collaborative ethnography. This project, ‘*Proyecto Postal Postpetrolera*’ (Postcards post‐petrol‐state), collected twenty postcards from Venezuelan migrants residing in Bogotá, Colombia, from 2021 to 2022. The result was the production of an overview of ‘snapshot’ moments from migrants' lives, taking the power for redaction away from the researcher and putting it into the hands of the participants themselves.

Of particular note is the unique time period in which this project took place – during the COVID‐19 pandemic years. The virus arrived in Colombia at the beginning of March 2020, with all borders to Venezuela closed on 14th March as a result (El Tiempo, 2020). Land borders would not be reopened until May 2021; however, the border with Venezuela remained closed due to a high perceived risk of contagion (Reuters, 2021). Lockdown extended from March to September 2020, though data collection for this project happened after that period. Colombia was one of only three countries in the world to impose a gender‐segregated quarantine, which was limited to the city of Bogotá. This system saw men and women only able to venture out of their houses on designated, opposite days; a situation that caused great difficulty for those not living with someone of the opposite sex, single parents, and trans men and women, to name a few (Irons, 2022a). This must be mentioned as migrants in particular are not necessarily living with their partners/families, who may be back in Venezuela or elsewhere. They, therefore, may have been particularly affected by this quarantine rule. Furthermore, at this already difficult time, Venezuelan migrants living in Colombia had limited access to healthcare services, depending on their legal status in the country (Stevenson and Guillén, 2023).

This article will explore the place of postcards as colonial mediums, and their contribution to the exoticisation of the ‘other’ as part of Empire imaginaries. The use of postcards in academia will be queried, to discuss where the present project sits in terms of the wider multimodal approaches using postcards in scholarship. The methodology employed within this project will be explored in depth, in order to both underscore the utility of such as a creative method within anthropology and migrant studies, as well as give a roadmap for future researchers to develop and improve upon this methodology in their own work. Having explored the background and methodology, the article will then turn to analysis of the postcards' content, highlighting the central themes within the snapshots of life described. Finally, it will be possible to conclude that the postcard medium offers a creative avenue for working with mobile migrant populations in a collaborative way, inviting other scholars to develop and improve upon the present methodology to explore new collaborative approaches with research communities, whether migrants or otherwise.

## The (Post)Colonial Postcard

Though the origin of the postcard is disputed, Gugganig and Schor (2020a) write that the most common reference is 1 October 1869 in Austria, with the ‘Golden Age’ of circulation peaking ‘in 1914 with 200 to 300 billion produced and sold postcards’ that year (2020a: 57). The authors argue that this coincided with colonialism and the explosion of global tourism, thereby perpetuating ‘exoticising imaginaries of far‐away places and people’ (Gugganig and Schor 2020a). It has been argued that the images used to adorn postcards were ‘charged’, and suggested a truth and objectivity about the colonised that obscured a more violent reality (Wehbe, 2006). Certainly, such snapshots of the colonies were notable particularly for their depictions of the people who lived in these so‐called ‘exotic’ destinations. As Haschemi Yekani and Schaper (2017: 609) note, racial differences were often stressed on picture postcards in the German (and other) colonies, thereby communicating the colonial order to the metropole back home. Photographic depictions of the people living under colonialism were a popular format, and have been much analysed for their contribution to subjective representations of those under colonial rule (van Eeden, 2009; Goldsworthy, 2010; DeRoo, 2013). Junge (2018: 168) argues that this acted as a catalyst for colonialism, with colonialist meaning ‘reinforced through the process of the postcard's delivery from colony to metropolis’. As such, postcards have been seen as ‘the perfect subjects through which to trace a colonial semiotics of representations and power’ (Vokes, 2010: 390), arguably making it difficult to separate the medium from colonial histories of oppression and representation. That said, it is important to remember that the postcards circulated during the colonial period would have been predominately sent between colonisers – not the colonised. Those who were depicted on the postcards did not have a say in their representation in many cases, and it is this understanding of postcards that the present project seeks to challenge.

Within this panorama, Colombia and Venezuela were both colonised nations until 1810 and 1811, respectively (Brown, 2012). Though no published scholarship exists on whether or not postcards in particular were used to communicate information about these countries to Europeans (Spain, in particular), there is more recent research hinting at Venezuela's history and use of postcards in the last century.

As a country possessing a wealth of varied and enchanting natural attractions, it is unsurprising that Venezuela previously attracted a number of European, North American and other international tourists annually. However, following Hugo Chávez's failed 1992 coup, tourism rapidly began to decline as would‐be travellers became concerned over security (Khol, 1992). The 2004 closure of six international airports due to safety concerns (Duarte et al. 2006) and the 2008 suspension of international cruise liner calls to Venezuelan ports over insecurity and economic turmoil (France24, 2023) concretised Venezuela's standing as a nation for tourists to avoid, with the tourism industry becoming ‘washed up’ as a result (Wilson Centre, 2023). Yet, it was not always this way. Once, before Caracas was granted the dubious honour of world murder capital (Kairuz, 2010), it was a city of interest whose skyline graced the frames of many tourist postcards. Carson (2016) eloquently describes coming across one such pack of Caracas postcards, now tattered and victim to a significant price mark‐down, presumably because the lack of tourists meant that there was no one to invest in the ritual of sending Caracas' vistas around the world. These nostalgic remnants of a bygone‐Venezuela are worth mentioning in the context of this project because it is important to know that Venezuela's tourism industry existed in living memory, and that it was accompanied by postcards. As such, the participants in the present project may well have come across such postcards of Venezuelan cities and attractions themselves, and be aware of the significance and use for this medium in a tourism context. That said, tourism is certainly not the only sphere in which postcards have been used in Venezuela. Postcards have also been linked to another of Venezuela's national pastimes – the pursuit of beauty and chasing the title of a ‘Miss’ (e.g. see Llinas, this volume). In her exploration of Venezuela's longtime national obsession with the beauty pageant, Ochoa (2014) describes how early‐day beauty contests produced postcards depicting contestants. The public would mail back the postcard showing their first choice contestant to the pageant committee (in this instance, a tobacco company). In a national context where aesthetic beauty is considered of utmost importance for social standing and advancement (Gulbas, 2017), the picture postcard's use in this area suggests that it too may hold some considerable significance for Venezuelans and their idea of nation.

Not limited to the national imagery of beauty, picture postcards have also been associated with a wide range of Venezuelan cultural developments, from political campaigns to infrastructural development. For example, Morrison (2023) explores the history of Venezuela's tram and railway infrastructure through the discussion of picture postcards depicting these advancements, concluding that the postcard served as a visual way to communicate economic development to a wide population, many of whom may have been illiterate. On the other hand, White (2023) discusses the ways in which postcards were used to suggest a ‘modern’ Venezuela of developed public works, flagship architectural projects, and infrastructure during the dictatorship of Marcos Pérez Jiménez in the 1950s. Here too, postcards offer a snapshot of a moment of Venezuelan history, used to depict a desirable image of the country to nationals and foreigners alike. In what follows, a new chapter of Venezuelan history is now offered, with migrant experiences discussed as they relate to each other, Colombia and their shared situation.

## Methods: Collaborative Ethnography via Mail

Within academia, postcards have been criticised as a mundane (Gugganig and Schor, 2020a) and banal (Ferguson, 2005) expression of popular culture, resulting in academic prejudice towards the medium (Gugganig and Schor, 2020a: 56) that may have caused them to be overlooked and even disparaged. However, as Gugganig and Schor (2020a) have recently argued so well, rethinking the postcard anew opens up its possibility for greater use within ‘ethnographic research, public anthropology, and applied community work’ (Gugganig and Schor, 2020a: 56). The authors suggest that in viewing the role of the postcard as extending beyond merely the frame and form within which it is received, we can also see research and community work as extending beyond the immediate and superficial framing. Indeed, postcards may be viewed as useful sources of evidence of the past (Quanchi, 2004) – whether that is centuries old colonial histories, or a recent and dynamic past of Venezuelan migration occurring within the last decade.

This project was borne from the desire and necessity to develop a creative and accessible way to collect data with and from Venezuelan migrants who were on the move, and/or living in precarious circumstances, during the pandemic. Though I had originally planned to conduct ‘traditional’, in‐person ethnographic fieldwork with migrant communities living in Colombia as part of a larger grant researching Venezuelan migrant health, the travel restrictions imposed by the global COVID‐19 pandemic made this impossible at the time. Owing to the digital poverty experienced by many migrants (Alam and Imran, 2015), virtual fieldwork methods may be difficult to employ when working with marginalised populations. This called for creative methodologies.

Postcards were chosen for a number of reasons. First, as discussed in the previous section, postcards have been contested as a (post)colonial medium due to their relationship with/in the European colonies. As part of a collaborative ethnographic approach, this project sought to challenge this view of a (post)colonial medium.

The postcards were to be used in an accompanying exhibition held at University College London in Spring 2024 (of which participants were informed). As such, it was necessary and important to utilise a visually appealing methodology so as to engage non‐expert publics with the stories and experiences written on the back (as opposed to, say, plain cards).

A call for participants was circulated amongst existing networks in Bogotá (developed through a wider project exploring healthcare access), with participants reimbursed for the postcard and stamp costs involved. Postcard collection took place from January 2021 to December 2022. The extended time period was to allow for time delays in the postal service delivery and to recruit as many participants as possible. One in‐country assistant helped to source participants and direct them to places where postcards and stamps could be purchased, and sent. Participants were requested to write a snapshot of their life in Bogotá. Participants were largely free to write what they saw as relevant, appropriate and important. A total of twenty postcards were successfully obtained.

In this article, I do not analyse the images on the postcards themselves, only the writing. Though a number of scholars have underscored the importance of the image itself, I would argue that this applies more to the colonial context, particularly when people are shown. None of the postcards sent showed humans or animals‐ they are all Bogotá city views, with the one exception depicting ‘Monalisa’ by Colombian artist Fernando Botero (who is from Medellin, but has a museum in Bogotá, perhaps explaining the postcard purchase). I chose not to analyse the chosen images for a number of reasons. First, there are limited postcard options in Bogotá with many being productions utilising Bogotá's ‘city brand’ (where the font of the city name features an oversized ‘A’ at the end of the word ‘Bogotá’, and an older version that utilises a stylistic font usually in yellow). City branding is a recent tourism phenomenon, especially in Latin America, and stems from an attempt to standardise and promote tourism to a country or region (Echeverri Cañas and Trujillo Gómez, 2014). It is, therefore, likely that participants chose Bogotá‐brand postcards because that is simply what is most on offer for purchase. Importantly, as described above, colonial uses of postcards focussed overwhelmingly on the image of the exoticised ‘other’ and/or place depicted. The other did not speak in the colonial postcard. Alternatively, this project and analysis will not dwell upon the postcard images themselves, but rather will focus on the narrative given by the migrants themselves in order to rework the use of the postcard in creative methodology. However, it is also necessary to acknowledge the limitations placed upon the project and subsequent analyses due to this methodological and analytical choice. Aside from the fact that people were unable to write a great deal, as a researcher, I was also unable to consult them or follow up on what they did chose to include. As such, whilst the postcards collected were numerous, there is limited in‐depth data on the individual experiences of each participant.

Participants noted various lengths of time living in Colombia. From the date of writing the postcard (between 2021 and 2022) these ranged from 3 years (4 participants), 4 years (2 participants), 5 years (3 participants), 6 years (1 participant), 9 years (1 participant), to 15 years (1 participant). Eight people did not mention the amount of time they had spent in Colombia. Out of those, only one specifically mentioned being in another country (Mexico) when the pandemic hit. As such, it can be concluded that the majority of the participants would indeed have been living in Colombia during the COVID‐19 pandemic.

Postcards were numbered according to when I received them (note: this might not necessarily correspond to when they were written). All postcards were coded with an identifying code from PC1‐20 for simplicity and easy identification of the translated quotes in the article text with selected attached postcard scans (e.g. PC8, PC9 and PC10). All translations were undertaken by me.

I am not the first to suggest the incorporation of postcards into research by any means. Indeed, postcards have been used in academic settings previously in various ways. For example, Gugganig and Schor (2020a) incorporated postcards into a project when Gugganig sent a call for colleagues and students to send postcards during fieldwork. Kamash (2017) collected visitor reflections on postcards following their viewing of a replica of the Palmyra arch in London, in their ‘postcards to Palmyra’ project. The postcard reflections allowed visitors the chance to feedback to the researcher and acted as a way for the researcher to collect data as well. Millman (2013) used postcards in a slightly different way for her ‘Postcards from the Cut’ study in Birmingham. For this method, she distributed six landscape postcards to local residents in order to illicit reflections on the changing environment.

Outside of academia, two Venezuelan artists have employed postcards in creative ways to reflect upon the ongoing situation. In 2014, US‐based Venezuelan artist Esperanza Mayobre developed the online showcase ‘Postcards from Venezuela’ (Mayobre, 2014). Mayobre uses photographs of contemporary Caracas such as queues for basic goods, revolutionary fervour, military police in battle formation and candles for the deceased, amongst other images, as her postcard frames. On the back of each, she included type‐writer anecdotes that were ‘stories she told to herself in order to explain her lost country’.

Colombia‐based Venezuelan photographer Vilena Figueira also utilised the postcard format to reflect upon the political crisis, this time innovatively incorporating both visual and audio methods, which she calls ‘augmented reality’ (Pedreáñez 2020). In her 2019 project *‘Postales de la memoria’* (Memory Postcards), upon request Figueira would send a postcard alongside a recording of a migrant reflecting nostalgically on Venezuela, alongside the gentle sound of waves. She argues that the geographical reference increases one's sense of nostalgia (Pedreáñez, 2020).

The present project was not informed by previous studies as such, but instead developed out of the necessity and desire to collect participatory data in a creative way given the contextual constraints, in addition to my own interest in the medium. Long before the inception of this project, postcards have played a role in my life. Being an anthropologist and often away from the UK, for the last decade, I have sent picture postcards of my travels to my two nieces in the hope that they one day would be able to use these to construct a visual history of their absent aunt's whereabouts. Unexpectedly, purchasing and mailing postcards taught me a great deal about the countries where I was visiting, particularly their bureaucracy and imageries most valued to represent the country for foreigners. The exercise eventually became as much a fun game for me as well as to leave evidence for young relatives that I had thought about them throughout their childhood.

When the pandemic hit the UK I was living alone, and like many people in that situation, felt lonely and quite bored. After noticing another academic's tweet about the scheme, I joined the online community ‘Postcrossing’, whereby users send picture postcards to strangers around the world, and in return receive postcards from other global users as well. Undertaking this lockdown activity sparked an idea – if I could receive information from international strangers talking about their daily lives, why not the Venezuelan migrants and their networks I was actually working with? I have since looked back over those ‘Postcrossing’ postcards received from 2020 to 2022, especially the two I have from Ukraine, and I cannot help but wonder how drastically the lives of those senders may have changed since they mailed me their daily life snapshots, talking about their hobbies and activities that are perhaps now mere memories to them as crisis has gripped their home country. As such, it is in that spirit that I proposed the present project to collect snapshots of Venezuelan migrant lives, creating a visual history of their experiences during one moment of time, in a fully collaborative way whereby the participants would have control over what information they felt was important to give, and that which they elected to omit.

In such an experimental project, there were undoubtedly a number of limitations and challenges. For example, it should be acknowledged that participation in this project would have been exclusionary to those who were illiterate/could not, or did not want, to find someone else to write the prose on their behalf. That said, Venezuela has a relatively high literacy rate at 99.14 percent for 2021 (Ortega and Rodríguez, 2008), so this was not envisaged as a key concern. There may have also been challenges when it came to navigating the Colombian postal system. As I mentioned above, in my many years of sending postcards from international locations, I am aware that this process can be far more complex than it perhaps should be. For example, finding the postal service, attaching the correct number of stamps corresponding to the destination and knowing where to deposit the postcard can prove difficult. However, advice was given on the cost to send a postcard to England – any combination of stamps may be used so long as a minimum corresponding monetary amount was used. That said, I received queries from my in‐country collaborator about whether or not participants should send postcards in a stamped envelope, for example, highlighting how the postcard medium may not have been completely familiar to all. The fact that postcards are sent as ‘open’ letters also points to a lack of confidentiality. Though participants could arguably not be publicly identified as they did not include addresses, some did include their names.

A limitation exists regarding data collection and validity within the research method itself. Namely, as participants were essentially unknown and anonymous, it was not possible to double check any information and/or illegible writing, for example. This could have its benefits, as participants would know that they would not need to engage with the project beyond the time spent writing and sending the postcard, so would not have been put off by potential future time investments. However, it also meant that some of the information was not included and left to the researcher to analyse by context and some conjecture (which was not the overall point of the exercise). Finally, many participants did not date their postcard so it is not possible to know exactly what time snapshot we are viewing (only that it is from 2021 to 2022). I hope that any future researchers intending to utilise the postcard methodology can learn from these limitations and develop their own methods accordingly.

## Snapshots of Life

### Migration Trajectories and Motives

A frequent opening line from postcard senders was the amount of time that they had been living in Colombia, at times with date‐specific additional information about when they left Venezuela – a moment in time that may be firmly seared into their memory. Amongst those who commented on their motives for leaving, a majority cited the political, economic and social circumstances that influenced daily life in Venezuela. Some participants sought a better life ‘in general’. For example, PC5 said that they ‘had to migrate here [Bogotá] in order to have a better quality of life for me and my family’. PC12, PC4, PC6 and PC16 all cited the Venezuelan economy writ large, writing ‘I am in Colombia because the Venezuelan economy, and the pandemic, totally affected my country. Many of us left to go to other countries’ (PC16; Figure 1.7); ‘I'm Venezuelan but I came to this country because of the current situation in my country, due to the inflation that has been rising. For the last 18 years now, I have been searching for a better quality of life’, and ‘I left Venezuela in 2019 because of the economic crisis. I came to Bogotá, Colombia, looking for work, for more food, and a better quality of life’ (PC16; Figure 1.7).

Others mentioned the effect that the economic crisis had had on their daily life, in particular their salaries. For example, PC7 says: ‘I lived in Guyana City, in Bolivar State. I graduated from University. I came here to Colombia because of the situation in Venezuela, the lack of food, the low pay at work…it was a very difficult situation’. PC10 describes how the economic crisis influenced their purchasing power: ‘When I left my country, it was because my salary did not cover the basics, even though I was working as a domestic maid and studying industrial hygiene and security’ (Figure 1.3). Though PC10 did not mention what those basics were specifically, both PC17 and PC13 point out hunger and lack of food as one of the consequences of the crisis:

I came to Colombia‐ Bogotá, and was motivated to migrate due to the strong crisis that my country is experiencing. In order to finish my studies, I had to go hungry and live through many difficulties (PC17; Figure 1.8).

I left Venezuela in 2017 due to the terrible circumstances. Now in my country everyone is hungry, we don't have any public services, nor transport, nor health services. I decided to come to Bogotá because it is the capital [of Colombia], and I wanted to look for a job (PC13; Figure 1.5).

PC13 highlights the lack of health service availability in Venezuela as a motivating factor for migration, which was corroborated by PC18 who wrote: ‘I left Venezuela in 2007 because of the lack of medication and food, and due to the revolution, and I established myself here in Bogotá’. The Venezuelan health system is currently under extreme stress, with a complete shortage of basic necessities (Page et al. 2019), exacerbated by the medical worker brain drain that the crisis has accelerated. It is no surprise that some participants saw this as an important factor to migrate. Importantly, both PC13 and PC18 mention specific circumstances that would have influenced their need to seek healthcare: PC13 was pregnant, and PC18 was living with HIV. As such, both participants would have experienced exponential need to seek medical treatment above and beyond the norm for migrants.

Only one participant elected to mention their method of transport in reaching Colombia, which was PC19 who stated an arduous journey as a *caminante*: ‘I arrived walking = 7 days’ (Figure 1.9). Without further context it is impossible to know why modes of arrival were omitted, however, it stands out that, as only one participant thought to mention it, perhaps this was not considered a noteworthy detail by the majority.

## Pandemic in Bogotá

As has been discussed above, the pandemic situation was particularly difficult for informal migrants living in Colombia, and some of the postcard entries comment on such a situation. The theme that reoccurred most frequently was the loss of employment, which may be difficult for a migrant to secure in the first place. For example, participant PC13 states simply that: ‘Because of the pandemic I lost my job. When I started that job, I was pregnant with twins, and my husband also lost his job’ (Figure 1.5). In this scenario, both the participant and her partner were left without work, whilst she was also struggling with a pregnancy during a health emergency. There is no further information about her health or whether or not she was affiliated with an EPS (Colombian health insurance) and/or receiving any health services at the time.

Another participant (PC16) mentioned losing their work due to the lockdowns restricting their ability to work as a street seller: ‘In 2020, the covid pandemic was one of the hardest times that I've experienced in Bogotá, because I was working as a mobile street seller, and we were not allowed to be in the streets because of the health emergency. I did not have money to eat nor to pay rent, I had to live from the kindness of the people that God put in my life’ (Figure 1.7). Working as an *ambulante* (mobile street seller) has been one of the more common forms of [self] employment for Venezuelans across Latin America due to its accessibility (Kersh, 2020), though this kind of work was one of the most affected due to the pandemic restrictions. PC17 also mentions the restrictions influencing their loss of employment (though they do not state their job), and further underscores the difficulty of accessing vaccinations due to ones status as a migrant: ‘Migrating changed my life. Then came covid, it was very difficult, I lost my job because we could not go out [of the house], I couldn't get food. It was difficult to start again from zero after the pandemic. The indifference towards giving vaccinations to Venezuelans [was difficult], but I made an effort and you can achieve anything’ (Figure 1.8).

However, one participant mentioned that they did not experience work difficulties. Though they do not indicate what kind of work it is they do, it could be surmised that loss of employment may have been context‐dependent: ‘I have been living in Colombia for 5 years. In that time, I have never been without work, thanks be to God’.

Indeed, not all reflections on the pandemic were necessarily negative. Alongside PC16 mentioning that kindness was shown to them from those that ‘God had put’ in their life, another participant noted that they received help: ‘During the pandemic I received help from charitable foundations and the church. Here the pandemic lasted for 2 years, during which time we were shut inside. But one day I will be able to go back to my country, once the dictator Nicolás Maduro is gone’.

There was one positive and reflexive comment on the pandemic situation, where PC9 describes the opposite of the aforementioned experiences with employment loss, instead finding a new role and stability during (thanks to?) the situation: ‘I came to Colombia to keep myself alive. With the Covid pandemic I found my job and more stability‐ bad things are not always bad!’ (Figure 1.2). Finally, one participant specifically noted the pandemic as a motivation to migrate to Colombia, seemingly because they thought they could try their luck in Bogotá where the situation might be more favourable than other countries in Latin America where they had lived: ‘When COVID‐19 hit I was living in Mexico, but I decided to leave because of the high mortality rates. I went back to Venezuela but only lasted three months there, so now I'm trying out Colombia’ (PC20; Figure 1.10).

### Daily Difficulties

Although the pandemic may have caused unique and considerable challenges to some of participants, their postcards highlighted other varied concerns and daily difficulties experienced that extended beyond simply the COVID‐19 health emergency. One such issue was the way that one participant felt that they had been treated by the local population: ‘I do want to highlight that there are a lot of xenophobic people here…I'd say out of 100 percent, 50 percent [are xenophobic] … they have a bad impression about Venezuelans’. Xenophobia and racism towards Venezuelans has been highlighted as a continent‐wide problem (Cortés‐Martínez, 2018; Irons, 2022b) and it is therefore perhaps no surprise that one participant elected to mention this in their snapshot experience. That said, PC1 wrote that they had experienced good reactions with the people in Bogotá, suggesting that not everyone may have had the same experience with locals: ‘Until now everything has been good and very positive. It has brought me things that I did not have in my country, like a little more financial stability and good people’.

Alongside reactions to/from the host country, other diverse challenges were also mentioned. For example, PC17 lists a variety of horrors experienced since arriving to Colombia, including assault and rape: ‘When I got to this country [Colombia] I had problems in finding work, my house was burgled, I was assaulted at work, I was raped, and I had to put up with all of this to bring my family here. I did it, and even though it was worth it, I suffered a lot’ (Figure 1.8).

Unrelated to the COVID‐19 pandemic, two participants mentioned their status as living with HIV, with PC19 mentioning their relatively high hospital admission rate and development of an opportunistic infection: ‘2020 = I was diagnosed with HIV/2021 = I was diagnosed with tuberculosis/ I have been hospitalised 5 times’ (Figure 1.9). The second participant to mention their HIV status gave more information, and highlighted a motive for migrating to Colombia as influenced by the dire Venezuelan health system: ‘I left Venezuela looking for a better experience thanks to the situation with [President Nicolás] Maduro, I wanted a better financial situation, especially because I am living with HIV I wanted better medication, and there [Venezuela] the health system is not very good’ (PC20; Figure 1.10).

It could be argued that the experiences mentioned above focussed predominantly on practical challenges and concerns. However, one participant instead chose to mention their emotional experience in being separated from relatives: ‘It was a very difficult experience for me because I had to go far away from all of my family when I was only 18 years old. Now it's been 5 years and 6 months without feeling any warmth from the people that I love the most’ (PC16; Figure 1.7). This postcard in particular highlights more enduring migrant experiences and the need for greater attention in mental health services, which is currently missing for many Venezuelan migrants (Salas‐Wright et al., 2022), particularly in the context of COVID‐19 in Colombia (Espinel et al. 2020).

## Hopes for the Future

Migration is not only experienced as a negative phenomenon, as some comments have suggested. A number of people accompanied commentary on some of the migratory struggles with their hopes and dreams for the future, underscoring that despite the political crisis, people are able to remain positive and hopeful for their lives. Some of these were specific goals about what they would like to achieve in Colombia. A number of participants saw their roles as helping others, for example, PC14 wrote: ‘I am an animal rescuer [rights activist] and veterinary technician. I migrated because of the country [situation] and also so I could carry on with my work. I would like to have my own animal refuge and be the voice for those that don't have a voice of their own’ (Figure 1.6), and PC15 reflected: ‘I am a physiotherapist; Venezuelan from Caracas, university graduate, with the plan to continue helping people to become physically rehabilitated’. PC2 also wanted to help people, though they saw this as happening back in Venezuela: ‘My dream for the future is to go back to my country and for everything there to be better…for me to have my own business, to finish my studies, and to have enough funds to be able to help a lot of people that are in need, and I would like to visit new countries’. The notion of a better Venezuela was echoed by PC10, who said that: ‘I still dream about my country being free…’ (Figure 1.3). PC11 had specific dreams about their future in Colombia, though they write that they have encountered obstacles in obtaining these: ‘I came to Colombia with many dreams for my life, like helping my family and children to achieve in life. I am an artisanal baker and I have a lot of experience in everything to do with breadmaking…I had a lot of dreams that I wanted to achieve bit by bit, like owning my own artisanal bakery. But with the rent, bills, food, and other expenses, it has become difficult for me’ (Figure 1.4). Finally, PC8 and P16 reflected a more generalised sentiment of hope for the future: ‘At the moment I am working as a salesgirl for a furniture store, but in the future, I see myself studying and having more adventures’ (PC8; Figure 1.1), and ‘In spite of everything that has happened I am optimistic about life, and I know that with effort and hard work I will be able to achieve all of my goals <3 …’ (PC16; Figure 1.7).

Such varied migrant dreams for the future represent the notion of ‘contingent hope’ put forward by Wilde et al. (this volume). The authors argue that this hope hinges upon the improvement of specific circumstances, which the postcard writers clearly underscore in various cases.

## Conclusion

Asking migrants to write a snapshot of their lives on the back of postcards gives people the chance to say what they consider most important when they have only a limited space to do so. This is very different from other social science methods, such as interviewing and surveys, whereby responses may be lengthy and/or participants may be required to respond to specific questions. In the project explored here, people only cited that which they considered to be key (or that which they considered to be the most interesting for a foreign audience). Despite the relative freedom of response, the life snapshots explored in this paper were surprisingly not too disparate when it came to the themes touched upon. Venezuelans living in Colombia have a shared experience, to an extent, and this project has underscored some of the principal concerns and thoughts of this population during the COVID‐19 pandemic years. That said, it is important to recognise some of the challenges associated with this creative methodology, and how they may have influenced the findings.

First, though the design intends to decolonise postcards through a fully collaborative method, ultimately it is the researcher that has applied thematic analysis to migrants' writing and attempted brief analyses of the same. Due to the data collection method and anonymity, it has not been possible to clarify any postcard content with the writers, leaving some questions unanswered. To some extent, this concern is mitigated in the accompanying exhibition to the project/ article, where postcards and their direct translations are presented without additional commentary from the researcher, allowing the audience to take the migrant's words at face value. For any future researchers planning to use the postcard method of data collection, I would therefore suggest that they too attempt to incorporate other ways in which the information can be presented with minimal interference from the researcher(s), if a decolonial methodology is sought.

Finally, I end with a post‐data collection anecdote that reflects an unforeseen outcome of the project. Since collecting the postcards, a number of migrants did actually contact me on Facebook (my name was on the call for participants, and I am known within some migrant networks in Bogotá anyhow), wanting to double check whether or not I had received their submission and asking for photographs of how the final exhibition looks. One person suggested that they felt inspired and wanted to send a postcard of Bogotá back to their family in Venezuela as well (though they did not trust the postal system with its safe arrival). Such participant engagement with academic research is rare – for example, I have never been asked to send academic papers to research participants. As such, I suggest that this kind of collaborative, visual methodology is successful not only as a means of creative data collection, but also as a way to develop and sustain horizontal relationships with participants, and involve them fully in research – from data collection to the final product(s).

## Example Post Cards